# Three-tube method and covered metallic stent for the treatment of anastomotic leakage after esophagectomy

**DOI:** 10.1186/s12876-020-01480-z

**Published:** 2020-10-07

**Authors:** Yonghua Bi, Zhengyang Wu, Mengfei Yi, Xinwei Han, Jianzhuang Ren

**Affiliations:** Department of Interventional Radiology, The First Affiliated Hospital of Zhengzhou University, No.1, East Jian She Road, Zhengzhou, 450052 China

**Keywords:** Anastomotic leakage, Esophageal stent, Drainage, Three-tube method

## Abstract

**Background:**

Anastomotic leakage is common and life-threatening complication after esophagectomy. The management of esophageal anastomotic leakage remains challenging. We aimed to determine the safety, feasibility and efficacy of three-tube method and covered metallic stent placement for the management of anastomotic leakage.

**Methods:**

Twenty-six consecutive patients with anastomotic leakage were treated using three-tube method and covered metallic stent and the medical records were retrospectively assessed. All patients received placement of abscess drainage tube, jejunal feeding tube and gastrointestinal decompression tube as well as esophageal covered stent, followed by continue abscess drainage, nutritional support and anti-inflammatory treatment. Tubes and esophageal stents will be removed once anastomotic leakage heals.

**Results:**

The procedure was technically successful in 23 patients (95.8%). A total of 31 covered stents were used. Esophageal stents and abscess drainage tubes were successfully removed from 14 patients. The median retention duration was 2.3 months and 2.6 months for stent and abscess drainage tubes, respectively. No perioperative death, esophageal rupture, massive hemorrhage, or other severe complications were observed during procedures. The abscess cavity had markedly decreased in 8 patients or disappeared in 16 cases. During follow-up, 8 patients died of cancer recurrence and 2 patients died of severe pulmonary infection. The 1-, 3-, 5-year survival rates were 60.1, 51.5 and 51.5%, respectively.

**Conclusion:**

Three-tube method and covered metallic stent placement is safe, feasible and efficacious for treatment of anastomotic leakage after esophagectomy.

## Background

Anastomotic leakage is a rare but life-threatening complication of esophagectomy for esophageal cancer or esophagogastric carcinoma [1, 2], with an overall mortality rate of 20 to 50% [3–6]. Contamination in the abscess cavity may corrode vessels and even result in a higher mortality [7]. Various conservative treatment protocols have been used for the management of anastomotic leakage over the past two decades, including the application of biodegradable fistulae plugs or fibrin glue, endoscopic transluminal drainage or clipping and metallic esophageal stent insertion [4, 8–10]. Despite these modalities, management of anastomotic leakage remains challenging and the optimal treatment protocol need to be determined [2, 4, 11].

In this study, three-tube method (abscess drainage tube, jejunal feeding tube and gastrointestinal decompression tube) and esophageal covered stent was used. We aimed to determine the safety, feasibility and efficacy of this protocol for the management of anastomotic leakage.

## Methods

### Patient selection

**Fig. 1:**
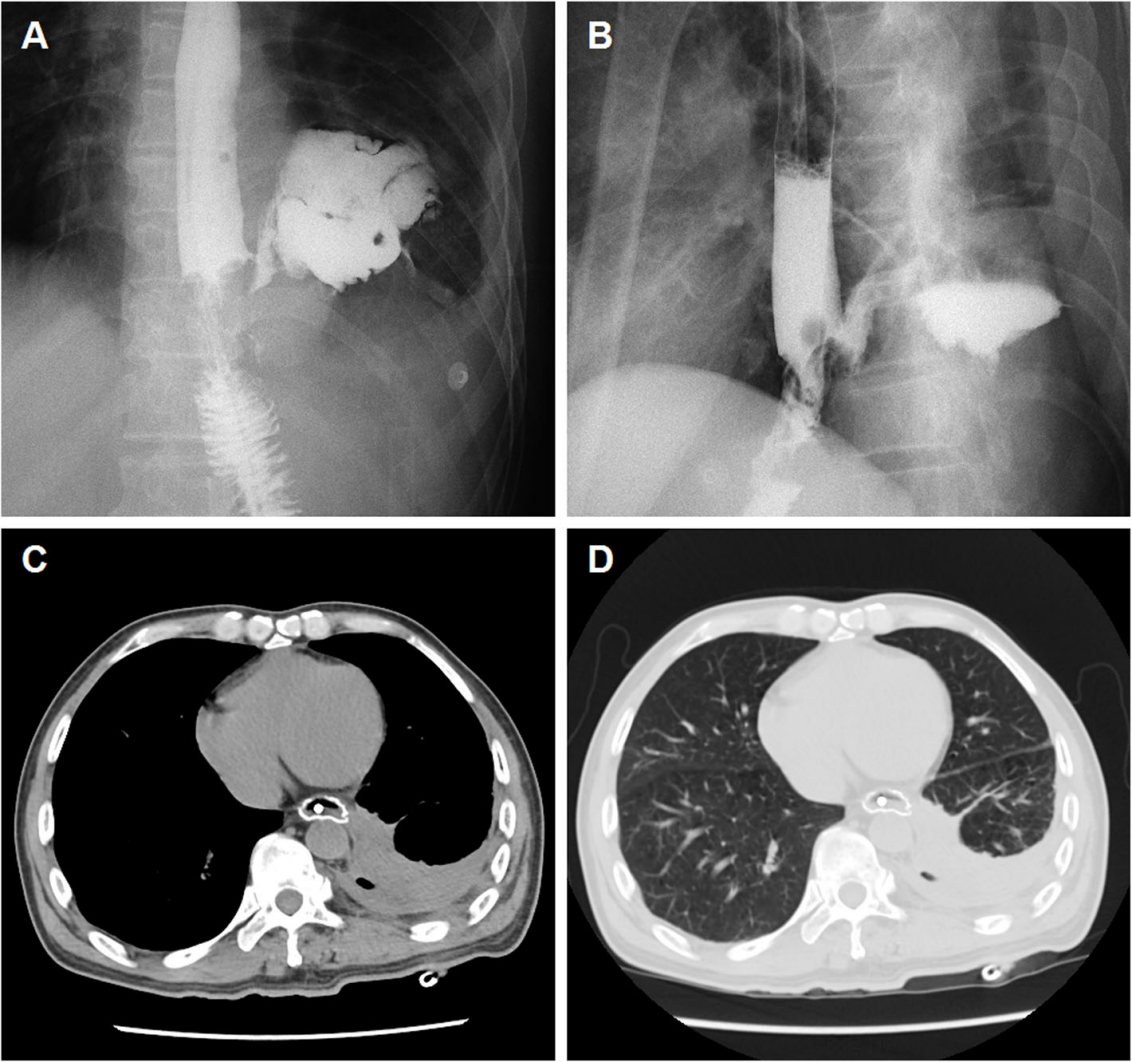
A 59-year-old man with anastomotic leak after esophagojejunostomy (case 24). **a**-**b** Esophagography showing an anastomotic leakage in the lower esophagus and irregular abscess in the pleural cavity. **c**–**d** Chest CT scan in the mediastinal and lung windows show mediastinal abscess and a small amount of pleural effusion before procedure

This study was approved by the Ethics Committee Board of Zhengzhou University First Affiliated Hospital. Informed consent was obtained from all patients. This study enrolled all patients with anastomotic leakage after esophagectomy who received three-tube method and esophageal covered stent placement in our institution between April 2011 and July 2018. The diagnosis of an anastomotic leakage was made based on esophagography (Fig. 1a-b) and chest computed tomographic scan (Fig. 1c–d). During the observation period, there were no changes in technique. Three-tube method was used due to financial difficulties or the position of the fistula was not suitable for stent placement. Patients were divided into 2 groups dependent on the size of the leak: small leaks (less than 6 mm) and large leaks (more than 6 mm).

### Three-tube method

All interventional procedures were performed under fluoroscopic guidance, local anesthesia and conscious sedation. The esophagus and pharyngonasal cavity was anesthetized with an oral lidocaine gel and a tetracaine spray, respectively. A 5-F cobra catheter was introduced through the outlet of anastomotic leakage and into the abscess cavity. The catheter tip was inserted into the distal end of the abscess cavity, followed by exchange with a 5-F pigtail or straight catheter (Cook Medical, Inc., Bloomington, IN). Continuous negative pressure suction was performed using a 20 ml syringe thereafter, with an appropriate pressure to drain the abscess at the same time avoiding excessive negative pressure in order not to cause iatrogenic injury and bleeding. We reduced negative pressure or stopped aspirating in case of bleeding. The abscess cavity was repeatedly rinsed with 100–200 ml of saline. A jejunal feeding tube and gastrointestinal decompression tube was inserted into the jejunum and gastric cavity, respectively. Enteral nutrition solution was infused via the jejunal feeding tube. Patients were allowed to resume oral intake once the leaks had been sealed by the covered stent and confirmed by contrast study. Broad-spectrum antibiotic treatment was performed before and after procedure.

### Esophageal covered stent placement

**Fig. 2:**
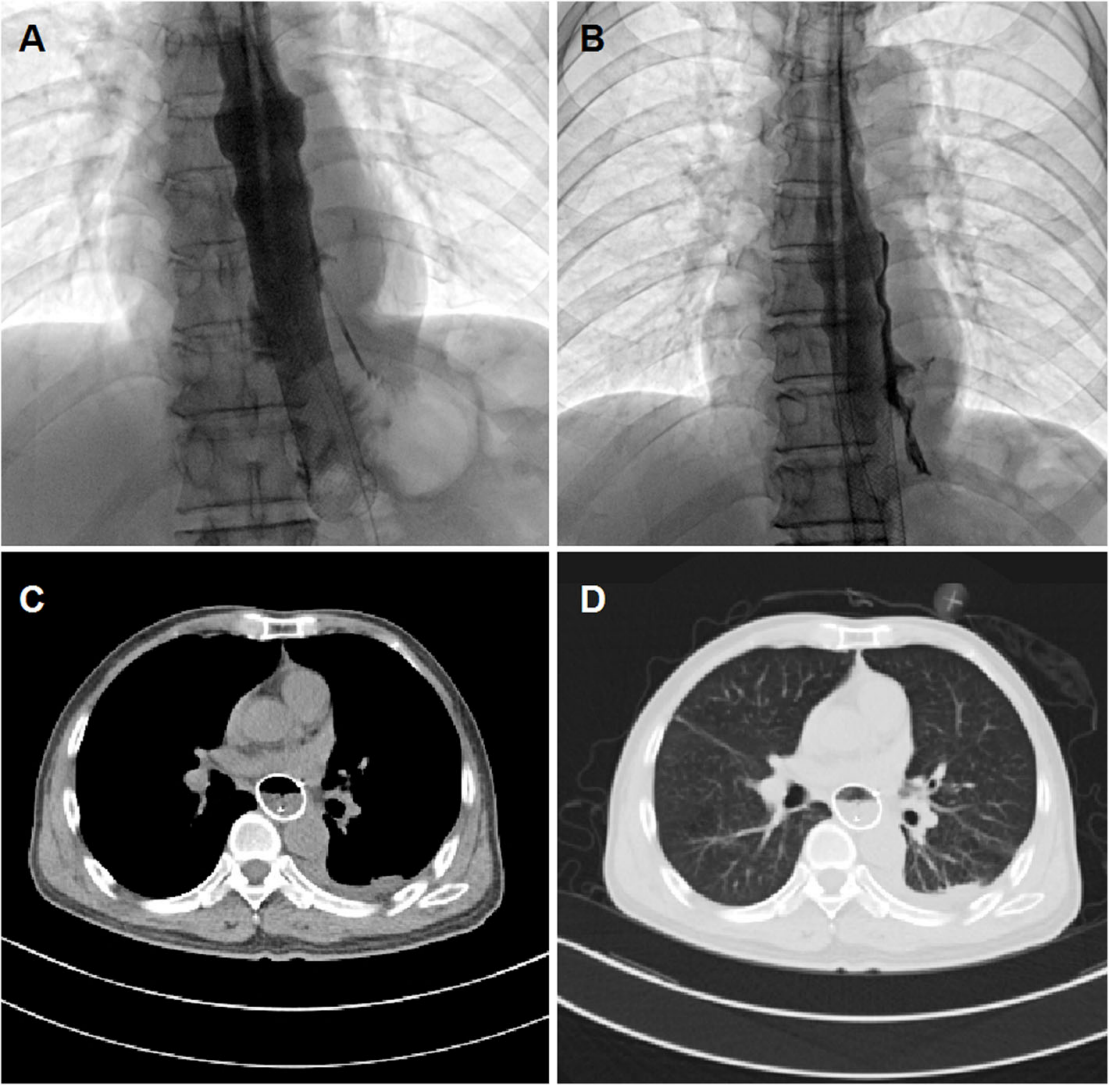
Three-tube method and covered stent placement for case 24. **a** After esophageal covered stent and drainage tube placement, esophagography shows that the contrast agent flows though the esophagus and stent with no leakage. **b** Esophagogram via drainage tube showing a decreased abscess cavity during follow up. **c**–**d** At 1.6 months after three-tube treatment and stent placement, a chest CT scan shows decrease in mediastinal abscess and pleural effusion

**Fig. 3:**
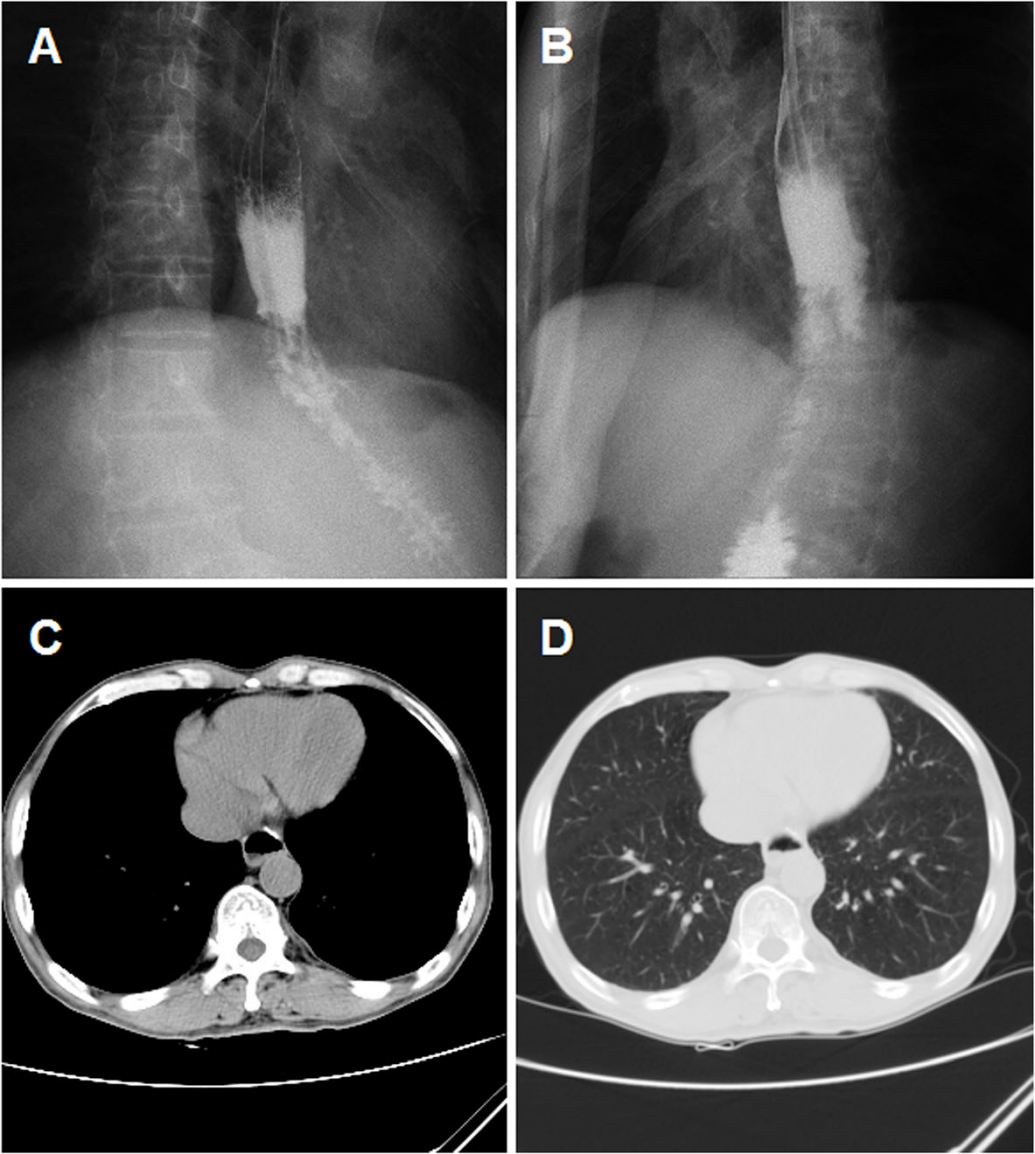
Examination after stent removal for case 24. **a**-**b** The drainage tube and stent were removed after procedure; esophagography shows that the contrast agent flows though the esophagus without any leakage. **c**–**d** The chest CT scan shows disappearance of mediastinal abscess, full expansion of the lungs with no pleural effusion

All patients received fluoroscopic placement of esophageal covered stent (Nanjing Micro-Tech Medical Company, Nanjing, China). The stent diameter ranged from 18 to 22 mm and stent length ranges from 70 to 160 mm. A 5-F catheter was inserted transorally into the gastric cavity and then a stiff guide wire was introduced. A covered stent was delivered via the stiff guide wire and then released carefully to block the leakage. A stent fixation line was used for fixation and stent adjustment. Leakage closure was confirmed by repeated esophagography (Fig. 2a-b). About 5 to 7 days after stent placement, chest CT and esophagography were performed again to study whether esophageal stent fit watertight, as well as the change of the abscess cavity and the position of abscess drainage tube (Fig. 2c-d). Stent adjustment or second stent placement were performed if esophageal stent does not fit watertight. The tube was adjusted to make sure effective drainage if necessary. The drainage tubes and stents were removed if complete disappearance of abscess cavity and full expansion of the lungs was confirmed by esophagography (Fig. 3a-b) and chest CT showed (Fig. 3c-d).

## Results

### General information

This study involved a total of 24 patients with anastomotic leakage, including 21 men and 3 women (Table 1). The age range of patients was 43 years to 76 years, with a median age of 59.5 years. Half of patients showed normal temperature after esophagectomy, the remained patients showed fever, with a median temperature of 38.6 °C (range: 37.5 to 40.0 °C). The median disease course before referral to our department was 3.0 months (range: 0. 3 to 12 months). The median interval between esophageal surgery and leakage was 0.4 months (range: 0.2–9.0 months). There were 16 cases of gastroesophageal anastomotic fistula and 8 patients showed anastomotic leaks after esophagojejunostomy.

**Table 1:** Patients characteristics of this study

**No.**	**Gender**	**Age (years)**	**Cause**	**Course of disease (Months)**	**Duration from surgery to leakage (Days)**	**Fistula type**	**Size (mm)**	**Location of fistula**	**Comorbidities**	**Pleural effusion**	**Atelectasis**	**Stent size ×**	**Complications**
1	Male	61	Esophageal cancer	1.5	9	Gastroesophageal anastomotic fistula	2.9	Lower part	None	A small amount	None	20 × 120	Stent migration
2	Male	52	Esophagogastric carcinoma	0.6	12	Gastroesophageal anastomotic fistula	6.5	Middle part	None	None	None	20 × 130;20 × 100	None
3	Male	68	Esophageal cancer	0.8	6	Gastroesophageal anastomotic fistula	7.0	Middle part	None	A small amount	None	20 × 70	Stent migration
4	Male	65	Carcinoma of gastric cardia	2.0	60	Gastroesophageal anastomotic fistula	8.0	Upper and middle parts	Esophageal stricture+coronary heart disease	None	None	20 × 120;20 × 120	Stent restenosis
5	Male	70	Carcinoma of gastric cardia	1.0	12	Gastroesophageal anastomotic fistula	8.4	Middle part	None	None	None	20 × 120	None
6	Male	53	Carcinoma of gastric cardia	2.0	150	Gastroesophageal anastomotic fistula	2.1	Middle part	Esophageal stricture	A small amount	None	20 × 120;18 × 160;18 × 120	Stent restenosis
7	Male	70	Esophageal cancer	6.0	171	Gastroesophageal anastomotic fistula	9.5	Middle part	Hypetension+Diabetes	None	Mild	18 × 120	None
8	Male	63	Gastric carcinoma	4.0	36	Anastomotic leak after esophagojejunostomy	4.8	Lower part	Esophageal stricture	None	None	20 × 100;20 × 100	Stent restenosis
9	Male	50	Esophagogastric carcinoma	3.0	30	Gastroesophageal anastomotic fistula	1.3	Lower part	Hypetension	A small amount	Moderate	18 × 100	None
10	Male	76	Esophageal cancer	1.0	6	Gastroesophageal anastomotic fistula	1.8	Middle part	Diabetes	None	None	18 × 120;18 × 120	Stent migration
11	Male	53	Gastric carcinoma	6.0	9	Anastomotic leak after esophagojejunostomy	5.1	Lower part	None	None	None	20 × 100	None
12	Male	58	Esophageal cancer	3.0	6	Gastroesophageal anastomotic fistula	5.5	Middle part	None	A small amount	Mild	18 × 120	None
13	Male	64	Carcinoma of gastric cardia	3.0	6	Anastomotic leak after esophagojejunostomy	9.6	Lower part	None	A small amount	Mild	20 × 100–20 × 40–20 × 40	None
14	Male	52	Esophageal perforation	4.0	12	Gastroesophageal anastomotic fistula	5.1	Lower part	None	Medium	Moderate	20 × 120	None
15	Male	58	Esophageal cancer	0.5	9	Gastroesophageal anastomotic fistula	8.8	Upper and middle parts	Diabetes	A small amount	None	20 × 80	Stent migration
16	Male	52	Esophageal cancer	5.0	15	Gastroesophageal anastomotic fistula	7.1	Middle part	None	A small amount	Mild	18 × 90	Stent migration
17	Male	60	Esophageal cancer	3.0	9	Gastroesophageal anastomotic fistula	9.3	Middle part	None	None	None	18 × 100	Stent migration
18	Female	51	Gastric carcinoma	11.0	270	Anastomotic leak after esophagojejunostomy	7.6	Lower part	Esophageal stricture	None	None	22 × 80–22 × 50–22 × 50	Failure in stent placement
19	Male	43	Carcinoma of gastric cardia	3.0	69	Anastomotic leak after esophagojejunostomy	6.7	Lower part	Esophageal stricture	Medium	Moderate	20 × 120	Stent migration
20	Male	54	Esophageal cancer	1.0	210	Gastroesophageal anastomotic fistula	7.7	Middle and lower parts	None	A small amount	None	18 × 140;20 × 120	Migration of stent and abscess drainage tube
21	Female	72	Gastric carcinoma	0.3	6	Anastomotic leak after esophagojejunostomy	4.1	Lower part	None	None	None	20 × 100	None
22	Male	65	Carcinoma of gastric cardia	1.0	15	Gastroesophageal anastomotic fistula	5.9	Lower part	None	A small amount	Mild	20 × 120	None
23	Female	65	Gastric carcinoma	6.0	18	Anastomotic leak after esophagojejunostomy	3.8	Lower part	Diabetes	None	None	20 × 140	Stent migration
24	Male	59	Carcinoma of gastric cardia	12.0	12	Anastomotic leak after esophagojejunostomy	5.2	Lower part	None	Medium	Moderate	20 × 140	None
**Adjustment/replacement time of stent**	**Adjustment/replacement time of abscess drainage tube**	**Abscess cavity**	**Removal of stent/abscess drainage tube**	**Retention of stent (Months)**	**Retention of drainage tube (Months)**	**Maximal body temperature (**°C**)**	**Leukocytes (×10**^**9**^**/L)**	**Neutrophil (%)**	**Other interventional treatments**	**Survival time after procedure (M)**	**Death cause**		
1	1	Disappear	Yes	1.6	14.6	Normal	7.7	72.5	None	67.1	Survive without symptom		
1	0	Disappear	Yes	1.5	2.1	Normal	4.5	64.9	None	24.5	Survive with slight reflux		
3	2	Decreased	Yes	10.5	10.6	39.0	7.4	76.3	None	19.9	Survive without symptom		
1	0	Decreased	No	1.8	1.4	38.5	8.7	62.7	Balloon dilation	6.6	Survive without symptom		
0	3	Disappear	Yes	2.2	2.3	38.9	11.1	92.0	Balloon dilation	12.3	Survive without symptom		
3	4	Disappear	Yes	3.2	5.9	38.6	12.8	88.2	Balloon dilation	17.3	Survive without symptom		
0	0	Decreased	No	6.8	6.9	38.1	16.5	86.2	PTCD	6.9	Died of cancer recurrence		
1	0	Disappear	No	1.0	1.0	Normal	3.9	45.4	None	6.8	Survive without symptom		
0	0	Disappear	Yes	2.4	1.4	39.0	20.0	84.7	None	14.4	Survive without symptom		
1	3	Disappear	No	6.0	2.2	Normal	6.0	85.2	None	6.0	Survive without symptom		
0	4	Disappear	Yes	2.1	5.9	38.6	11.5	93.1	None	8.1	Died of cancer recurrence		
0	0	Decreased	No	6.1	6.1	40.0	11.0	84.8	None	6.1	Died of cancer recurrence		
0	2	Decreased	No	2.9	2.9	39.5	7.5	84.4	None	2.9	Died of cancer recurrence		
0	2	Decreased	Yes	4.0	3.3	Normal	8.4	59.0	None	147.6	Survive without symptom		
2	2	Disappear	Yes	8.4	11.4	Normal	17.6	94.2	None	23.9	Survive without symptom		
3	0	Decreased	No	2.8	2.8	Normal	12.0	75.6	None	2.8	Died of pulmonary infection		
3	0	Disappear	Yes	1.0	0.4	Normal	11.5	91.6	None	1.4	Died of cancer recurrence		
0	2	Disappear	No	0.0	1.4	Normal	10.3	84.6	Balloon dilation	1.3	Died of cancer recurrence		
1	2	Disappear	No	1.3	1.3	37.5	15.6	89.3	None	1.2	Died of pulmonary infection		
2	3	Decreased	No	14.3	18.6	Normal	14.0	86.0	None	14.3	Survive, but the fistula wasn’t healed		
0	2	Disappear	Yes	0.6	0.6	Normal	12.9	90.1	None	2.1	Died of cancer recurrence		
0	6	Disappear	Yes	2.4	2.9	Normal	6.4	80.0	Bilateral ureteral stent implantation	7.6	Survive without symptom		
1	0	Disappear	Yes	1.5	1.5	38.3	23.5	95.4	PTCD+stenting in colon	19.9	Died of cancer recurrence		
0	0	Disappear	Yes	1.1	1.2	38.5	19.9	92.3	None	60.4	Survive without symptom		

*PTCD* percutaneous transhepatic cholangial drainage

### Interventional procedure outcomes

Three-tube method was performed successfully for all patients (100%), only 1 patient failed Y stent placement due to complete occlusion (95.8%). A total of 31 esophageal covered stents were placed, with a median diameter of 20 mm (range: 18–22 mm), median length of 120 mm (range: 70–160 mm). Stent placement was successful in the remaining 23 patients, with satisfactory expansion and appropriate position. For those patients, all anastomotic leakages were completely blocked and all stents fit watertight after covered stent placement confirmed by immediate post procedural esophagography. Three patients were in need of external thoracic drainage. All patients showed a reducing amount of drainage fluid, approximately 20 to 250 mL per day. Body temperature returned to normal within 2 weeks after the interventional treatment for patients with fever.

### Complications

No perioperative death, esophageal rupture, massive hemorrhage, or other severe complications were observed during procedures. No intensive care was needed due to a worsening condition after the procedure. No necrosis due to negative pressure effects on the esophageal wall was observed after stent placement. Stent migration, the most common complication, was found in 9 patients, with a migration rate of 39.1% (9/23). Three patients showed stent restenosis, with a restenosis rate of 13.0% (3/23). All migrated or restenosed stents were adjusted or replaced for 1 to 3 times (median: 1.0 time). Migration of abscess drainage tube was found in 1 patient. The abscess drainage tube was adjusted or replaced for 0 to 6 times (median: 2 times).

### Follow-up

**Fig. 4:**
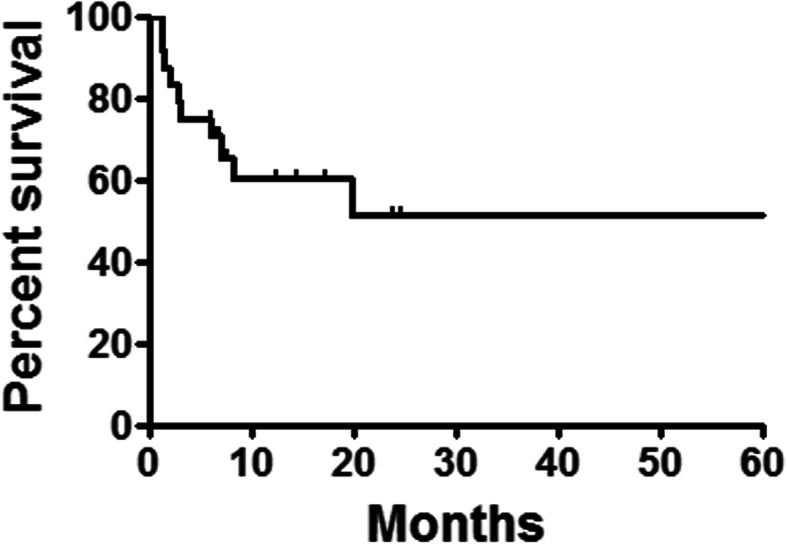
Survivals follow up. The 1-, 3-, 5-year survival rates were 60.1, 51.5 and 51.5%, respectively

All patients were successfully followed up, with a median time of 7.9 months (range: 1.2–147.6 months). Chest CT and esophagography showed that the abscess cavity had markedly decreased in 8 patients or disappeared in 16 cases. A higher rate of abscess cavity resolution was found in smaller leaks compared to that of large leaks (6/12). However, this different was not statistically significant (*P* = 0.11), due to small sample size. During follow up, esophageal stents and abscess drainage tubes were successfully removed from 14 patients. The remaining patients refused to remove stent due to heavy financial burden and fear of possible risks of removal. The median retention duration was 2.3 months (range, 0–14.3 months) for stents and 2.6 months (range, 0.4–18.6 months) for abscess drainage tubes, respectively. To this date 14 patients are still alive, with 12 patients returning to their normal daily activities of living and symptom free and 1 patient with slight reflux which is not requiring any treatment. During follow up, 8 patients died of cancer recurrence and 2 patients died of severe pulmonary infection. The 1-, 3-, 5-year survival rates were 60.1, 51.5 and 51.5%, respectively (Fig. 4). A higher rate of 1-year survival rate (70.7%) was found in small leaks than that of large leaks (48.6%). However, this different was not statistically significant (*P* = 0.31).

## Discussion

Management of anastomotic leakage is challenging for patients received esophagectomy The mortality is high, however, the optimal treatment still need to be determined [2, 4, 11]. Various conservative treatment protocols have been reported for the treatment of anastomotic leakage over the past two decades, including the application of biodegradable fistulae plugs or fibrin glue, endoscopic transluminal drainage or clipping and metallic esophageal stent insertion [4, 8–10]. Surgical repair of the anastomotic leakage is the traditional protocol, such as, esophagectomy or thoracotomy and repair [12]. Despite of the advances in surgical technique, the overall mortality remains as high as 20 to 50% [3–6]. Nowadays, treatment of choice in the first line is an endoscopic approach.

Esophageal stents were initially served as a palliative treatment for patients with esophageal carcinoma. Currently, esophageal stents have been used to treat esophageal benign diseases [13–17]. Metallic stents are usually used for treatment of tumor stenosis or esophageal perforation [18, 19]. Successful and effective management of anastomotic leakage needs prompt elimination of contamination by covered stent placement, and adequate drainage of the abscess cavity. We present 24 consecutive patients treated with three-tube method and covered stent placement for anastomotic leakage. Our clinical outcomes indicated that this interventional method can easily be performed under fluoroscopic guidance. No perioperative death was observed, which is lower than previous reports [6, 15, 16, 20]. After covered stent placement, the leakage is still allowed to continuously drain fluid via abscess drainage tube. In our study, all patients received continue abscess drain for a median duration of 2.6 months. Drainage of an abscess cavity is also possible percutaneously under CT scan control and it is generally easier for the subsequent follow-up. In this study, three patients were in need of external thoracic drainage. Compared with endoscopic drainage, radiologic drainage can be performed via the leaks without the need of percutaneous puncture.

The duration between esophageal surgery and leakage diagnosis is essential for the clinical outcomes [12]. The median interval between surgery and leakage was 0.4 months. The clinical outcomes were favorable; abscess cavity was markedly decreased in 8 patients and disappeared in 16 cases. Treatment results may be related to the size of the leak, and effective results of biodegradable fistulae plugs or fibrin glue application are generally observed only in small leaks.

Certain complications can be found in our interventional protocol. Stent migration is a common complication, especially in patients without esophageal stricture [21, 22]. All esophageal stents used in our study were coved ones, which may account for high rate of stent migration. Nine patients showed stent migration and were adjusted or replaced for 1 to 3 times. Only 1 patient showed migration of drainage tube, however, drainage tubes were regularly adjusted and replaced for 0 to 6 times to achieve effective drainage during follow up. The abscess drainage tubes were adjusted or exchanged for a median time of 2 times. Besides, esophageal stents and abscess drainage tubes were successfully removed from 14 patients, without difficulties of removal or severe complications. Recovery lines in the proximal end of stent are used for stent fixation to avoid stent migration, and for the adjustment or recovery of the migrated stent. Appropriate size of stent should been used, considering that small size of stent is prone to migrate. Of course, clips and stent with flaps can be used to reduce the migration rate.

There were some limitations. This was a retrospective study with relatively small number of enrolled patients. The esophageal stents and abscess drainage tubes needed adjusted or replaced repeatedly during follow up. BMI data had not collected previously considering that BMI may be not closely related to our treatment. We had not measured how much negative pressure and the study interval was long.

## Conclusions

Three-tube method and covered stent placement can be considered a safe and effective alternative to operative treatment for anastomotic leakage after esophagectomy. Combined interventional protocol with additional supportive therapy is useful to achieve good clinical outcomes.

## Data Availability

For further details, the corresponding author can be contacted.

## Abbreviations

CT: Computerized tomography
IQR: Mean ± interquartile range
